# Sequencing and annotation of the *Ophiostoma ulmi* genome

**DOI:** 10.1186/1471-2164-14-162

**Published:** 2013-03-12

**Authors:** Shima Khoshraftar, Stacy Hung, Sadia Khan, Yunchen Gong, Vibha Tyagi, John Parkinson, Mohini Sain, Alan M Moses, Dinesh Christendat

**Affiliations:** 1 Department of Cell & Systems Biology University of Toronto, Toronto, Canada; 2 Center for Analysis of Genome Evolution and Function, Toronto, Canada; 3 Departments of Biochemistry and Molecular Genetics University of Toronto, Toronto, Canada; 4 Molecular Structure and Function Hospital for Sick Children, Toronto, Canada; 5 Centre for Biocomposites and Biomaterials Processing, Faculty of Forestry, Toronto, Canada

## Abstract

**Background:**

The ascomycete fungus *Ophiostoma ulmi* was responsible for the initial pandemic of the massively destructive Dutch elm disease in Europe and North America in early 1910. Dutch elm disease has ravaged the elm tree population globally and is a major threat to the remaining elm population. *O. ulmi* is also associated with valuable biomaterials applications. It was recently discovered that proteins from *O. ulmi* can be used for efficient transformation of amylose in the production of bioplastics.

**Results:**

We have sequenced the 31.5 Mb genome of *O.ulmi* using Illumina next generation sequencing. Applying both *de novo* and comparative genome annotation methods, we predict a total of 8639 gene models. The quality of the predicted genes was validated using a variety of data sources consisting of EST data, mRNA-seq data and orthologs from related fungal species. Sequence-based computational methods were used to identify candidate virulence-related genes. Metabolic pathways were reconstructed and highlight specific enzymes that may play a role in virulence.

**Conclusions:**

This genome sequence will be a useful resource for further research aimed at understanding the molecular mechanisms of pathogenicity by *O. ulmi*. It will also facilitate the identification of enzymes necessary for industrial biotransformation applications.

## Background

Ophiostomoids are the most common Mycelial fungi associated with bark beetles. Within this group is *Ophiostoma ulmi,* the causative agent of the first incident of one of the most destructive plant diseases, Dutch elm disease (DED), starting from the early 1910s in Europe and North America [1]. The far more aggressive species *Ophiostoma novo-ulmi* accounts for a second DED pandemic which was initially recorded in Britain and is believed to have spread to North America from Central Europe in the early 1940s [2,3]. As a consequence of both occurrences, the majority of mature Dutch elm trees were destroyed in North America, Europe and central and southwest Asia. These incidents had tremendous economic impacts on the global forestry and horticultural industries. Unfortunately, bark beetle disease is still a major threat to the remaining North American elm trees, especially in Western Canada yet very few resources are directed towards their control because the molecular basis for *O. ulmi* pathogenicity is still not understood [4-6].

DED is a result of the bark beetle attacking the bark of trees and penetrating into the soft tissue where they feed on nutrients within the phloem [7,8] Concurrently, Ophiostoma fungi are transferred by the beetles to the phloem network where they colonize on the soluble tissues and block the transport of nutrients and water throughout the trees. This colonization of Ophiostoma and competition for nutrients produces an irreversible disease phenotype in mature elm trees, leading to the eventual death of these trees. The two subgroups *O. novo-ulmi* and *O. ulmi* are classified as aggressive and non-aggressive, with *O. novo-ulmi* being the aggressive species [9-12]. Further, they have distinct biological differences, such as growth rate, temperature optimum and colony appearance. As expected, the non-aggressive species is a weak elm pathogen in contrast to the aggressive *O. novo-ulm*i species, but both species produce characteristically distinct levels of the toxin protein, cerato-ulmin which is important in protecting infectious propagules from desiccation and is thought to act as a parasitic fitness factor for the organism [13-18].

Interestingly, attempts to cross *O. novo-ulmi* with *O. ulmi* are frequently rejected by the aggressive *O. novo-ulmi* female fungi. The hybrid progenies from successful crosses are usually of low competitive fitness and show low growth rate, decreased pathogenicity, low cerato-ulmin protein production and usually sterile females [19,20]. The differences in biological traits between the aggressive and nonaggressive species point towards incompatibility in their genomic composition. Thus, the genome sequence of these two species would present a unique opportunity for comparative analysis to understand the basis for their pathogenicity, and genomic incompatibility.

Biotransformation strategies have been developed for the use of *O. ulmi* protein extracts in the production of thermoplastic materials. While the protein identities and composition of such mixtures remain uncharacterized due to a lack of an available genome sequence, the quality and consistency of the thermoplastics produced is sufficient for the manufacturing of certain products [21]. Such approaches are highly attractive from an environmental standpoint for the use of renewable resources in manufacturing processes. Therefore, the application of *O. ulmi* has gained tremendous interest in recent years and has resulted in multiple patents. Unlike white-rot and brown-rot fungi (Phanerochaete) whose genomes are sequenced and annotated and are used in a plethora of commercial applications [22], *O. ulmi’s* recent emergence in commercial application was based solely on its ability to modify plants polysaccharides. This rather coarse approach in utilizing *O. ulmi* protein extracts in polysaccharides biotransformation is restricted because of a lack of a sequenced genome. Similar to the Phanerochaete fungi, the sequencing of *O. ulmi* would provide tremendous opportunities for its use in industrial applications.

Here, we report a first draft of the genome sequence and annotation of *Ophiostoma ulmi* strain W9. To validate the quality of the gene annotations, we employed EST sequences from *Ophiostoma novo-ulmi*[23], mRNA-seq sequences, and ortholog sequences from three other fungi, *Grosmannia clavigera*[24,25] and two model organisms *Neurospora crassa*[26] and *Saccharomyces serevisiae*[27]. We found that multiple lines of evidence support the quality of the gene annotations. An initial search for genes involved in the pathogenicity of the fungus was performed. The availability of the complete genome sequence should facilitate further studies of *Ophiostoma ulmi* and may be an important step toward development of molecular strategies for controlling DED.

## Results

### Genome sequence assembly and annotation

Using a whole genome shotgun sequencing strategy, we sequenced the *O. ulmi* genome to an average coverage of 200× by paired-end sequencing (see Methods). A 31.5-Mb genome sequence was obtained by assembling approximately 164 million Illumina reads. Genome statistics are given in Table 1.

**Table 1:** **General characteristics of the *O. ulmi *genome**

	
Size assembled genome (Mb)	31.5
GC content overall (%)	50.02
GC content (coding) (%)	57.8
Protein coding genes	8639
Gene density (genes/kb)	1/3642
Average gene length (bp)	1854
Average number of introns per gene	1.14
Median intron size (bp)	67
Median exon size (bp)	395

To annotate the genome, we combined an *ab initio* method with a comparative gene-finding approach. First we obtained gene models using the *ab initio* method GeneMark ES-v2.0 [28]. The main motivation for this approach was that it takes as input data the genomic sequence alone and requires no other input data such as training sets of known genes from *O. ulmi* or genes from other species. In order to identify conserved genes that were not found by the *ab initio* method, we used Exonerate [29] to align protein sets from *N. crassa*[26] and *G. clavigera*[24,25]. We chose *N. crassa* and *G. clavigera* because they are fully sequenced and annotated and both organisms are closely related to *O. ulmi*. This gene prediction strategy yielded 8639 genes in the *O. ulmi* genome, covering 45% of the total genome. In our final gene set, the vast majority of gene models (8553) were taken from the *ab initio* gene-finding method because most of them have start and stop codons in comparison to genes predicted by Exonerate. Comparative genome analysis revealed that approximately 71% of the annotated genes have orthologs from at least one of the three species, *G. clavigera*, *N. crassa* and *S.cerevisiae*.

**Figure 1:**
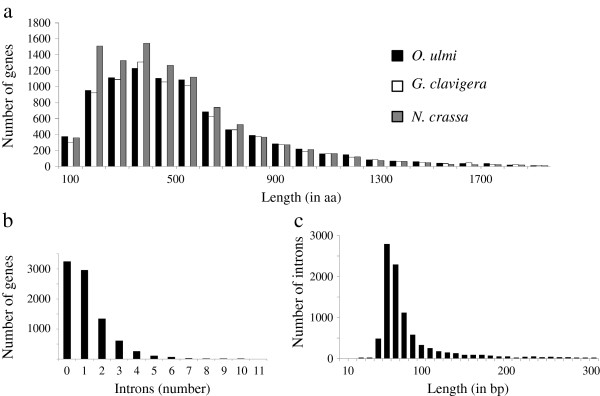
**Overview of genome annotation statistics. a**) the distribution of the lengths of the genes of the *O. ulmi* with *G. clavigera* and *N. crassa*. **b**) the number of introns in each of the predicted genes in *O. ulmi*. Most of the predicted genes have either zero or one introns and a few of them have more than 5 introns. **c**) distribution of the length of the introns of the gene models. The majority of the introns are 60 bp to 200 bp and 3% of them are between 200 bp and 300 bp. Almost 8% are longer than 300 bp but are not shown in the figure for the purpose of clarity.

We next compared the features of the *O. ulmi* genome with those reported for *G. clavigera* and *N. crassa*. The G + C content for all three species is approximately 50%. The number of predicted protein-coding genes for *O. ulmi*, *G. clavigera* and *N. crassa* varies considerably (8639, 8314 and 10,082, respectively) but the average gene density of *O. ulmi* is one gene per 3.6 kb which is very similar to that of *G. clavigera* and *N. crassa*, having one gene per 3.5 kb and one per 3.7 kb respectively. The average gene length of *O. ulmi* is 1.85 kb, slightly higher than 1.673 kb for *G. clavigera* and 1.67 kb for *N. crassa* (Figure 1a). The mean number of introns per gene is 1.14 for *O. ulmi*, which is somewhat less than 1.86 for *G. clavigera* and 1.7 for *N. crassa* (Figure 1b). The sizes of introns in *O. ulmi* are similar for the three fungal species compared (Figure 1c).

### Identification of *O. ulmi* gene orthologs and phylogenetic analysis

**Figure 2:**
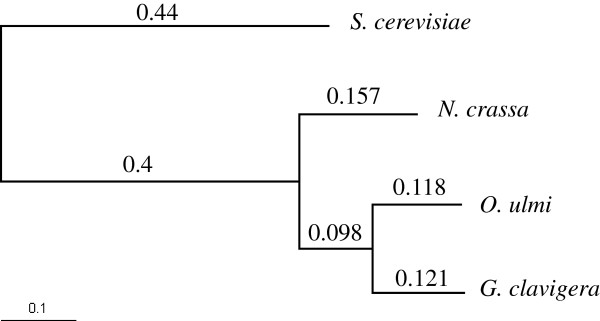
**Phylogenic tree.** Phylogenetic tree between *O. ulmi* and three other species *G. clavigera*, *N. crassa* and *S. cerevisiae*. Branch length represents the phylogenetic distances in substitution per site.

To evaluate the evolutionary relationships between *O. ulmi* and other fungi where a complete genome sequence was available, we identified orthologs of *O. ulmi, G. clavigera, N. crassa, and S. cerevisiae*, using Inparanoid [30]. Applying this approach we identified 5784 ortholog with *G. clavigera*, 5517 with *N. crassa* and 2483 with *S. cerevisiae*. Phylogenetic analysis was performed with the amino acid sequences of the 2215 genes for which we have a one-to-one ortholog relationship for each species with *O. ulmi.* We computed the likelihood of four possible trees with *S.cerevisiae* as the outgroup using PAML [31]. The tree, shown in Figure 2, was found with 90% bootstrap support for more than 80% of the orthologous groups. To estimate the distances between species, we took the median value of each branch length (in substitutions per site, Figure 2) from the 2215 genes for which we have a one-to-one ortholog relationship for each species. The total distance between *G. clavigera* and *O. ulmi* is more than 0.2 subs. per site, which is consistent with a previous analysis on two genes [32]. Our analysis indicates that *O. ulmi* is a representative of a divergent clade for which, to our knowledge, no complete genome sequences currently exist.

### Validation of genome annotation

In order to provide support for our gene models, we employed three sets of data. First, we used the published expressed sequence tags (EST) library from *O. novo-ulmi*[23]. Using est2genome model with default parameters from Exonerate [29], we mapped the EST data to the *O.ulmi* genome. We then looked for gene models that overlapped with positions in the genome mapping back to the EST data. We found that 91% of the gene models had expression evidence from EST data.

**Figure 3:**
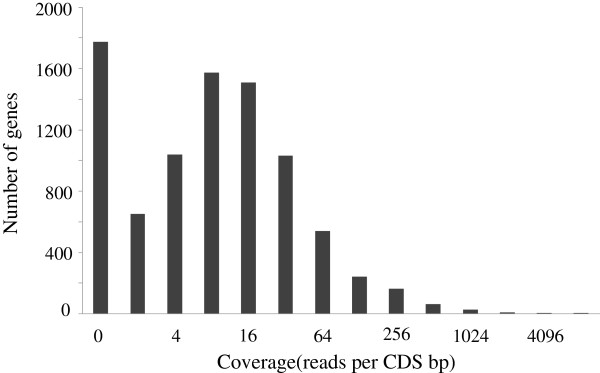
**Distribution of coverage for the coding regions of the gene models.** The coverage for the coding regions of the predicted genes is shown. Almost 80% have the coverage greater than 1.

We next compared our annotation to *O. ulmi* mRNA-seq read data we generated. Mapping mRNA-seq reads back to the genome using a short read aligner (Bowtie [33]), we found the average coverage to be 30.4 reads per base pair. Computing the coverage for coding segments of the gene models (See Methods), we found the coverage in coding regions to be 48.1 (reads per coding region base pair). Thus, the average coverage is 58% higher within our predicted coding regions than in the genome overall, providing evidence that our gene predictions are enriched for *bona fide* genes in *O. ulmi* (Figure 3). Despite this high average coverage, approximately 20% of the gene models have coverage of less than 1. We speculate that this may result from either lack of expression in the condition that the mRNA was extracted, or errors in gene prediction.

**Figure 4:**
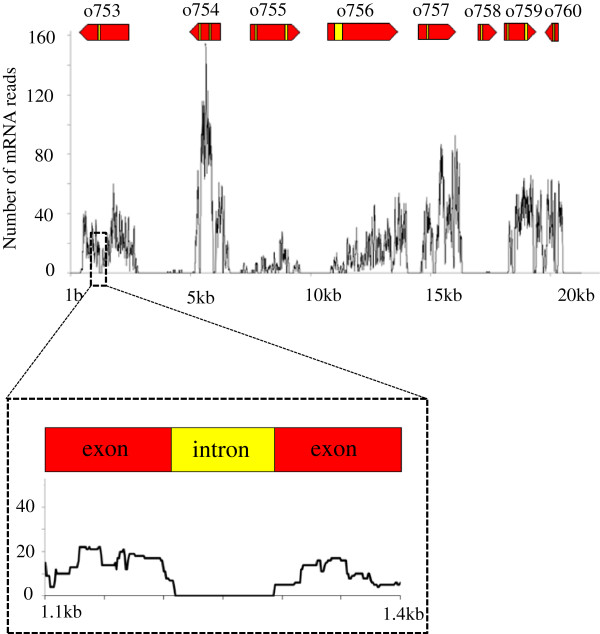
**Examples of mRNA-seq coverage.** The number of mRNA-seq reads mapped is plotted as a function of genomic coordinate. Eight predicted genes are displayed by red arrows, (o753 to o760). Yellow sections inside the red arrows refer to the intron parts of the gene models. A zoomed version of the area in which the intron appears is given in the box below the figure.

A typical distribution of the mRNA-seq reads for a region of 20 kb in the genome is shown in Figure 4. Gene o753 exemplifies a high-confidence gene prediction because there are approximately 20 reads covering it and the coverage drops to zero at the predicted intron-exon boundaries. On the other hand, gene o758 is an example of imperfect prediction because there is evidence for expression outside of the predicted coordinates.

As part of our evaluation of the gene model predictions, we have also included gene models with orthologs to at least one of the three other comparative species *G. clavigera*, *N.crassa* and *S. cerevisiae*. Significantly, 70.4% of our predictions were found to have 1-to-1 orthologs in other species and given that orthologs are often similar in function [34], this suggests that most of our predicted genes can be assigned a function based on comparison with genes of other well-studied organisms. This further supports the assertion that the gene models described here are accurate.

**Figure 5:**
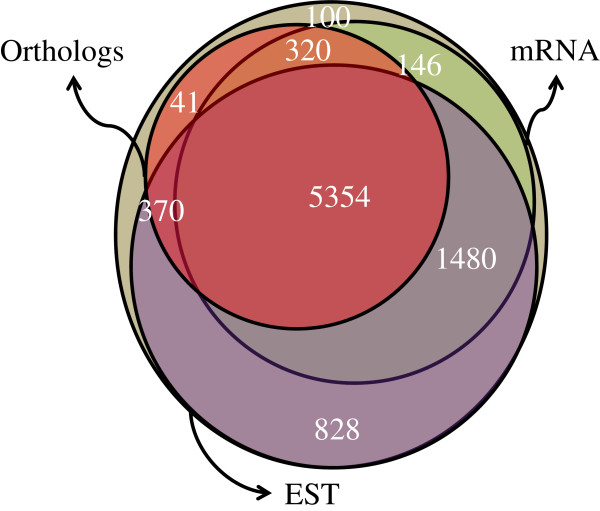
**Summary of genome annotation validation.** The biggest circle shows the total number of gene models predicted and every other circle represents a subset of gene models that are supported by any of the three types of evidences. The gene models that have evidence from EST data from *O. novo-ulmi* are referred to by EST data in the figure, those having ortholog genes from at least one of the mentioned three species referred to by Orthologs and those with evidence from mRNA-seq data from *O. ulmi* referred to by mRNA-seq.

Overall, ~99% of the gene models have at least one type of evidence with ~62% of them having all three types of evidence (Figure 5). This analysis indicates that our draft genome assembly and gene annotation is of high quality.

### Protein domain analysis

Previous studies suggested that specific genes of a pathogen are important for its pathogenicity [35,36]. For the fungi *O. ulmi* and the closely related species *O. novo-ulmi*, it was suggested that a hydrophobic protein Cerato-ulmin and a colony type gene col1 were directly correlated to the fungi causing DED in elm trees [37-39]. To check for the occurrence of other related genes (homologues), we searched our predicted gene set using these genes as queries. For both the hydrophobic protein Cerato-ulmin and col1, high confident single matches are found using Blast-2.2.25 [40] (e-value 6e-51 and 5e-86 respectively). While consistent with the accuracy of our gene predictions, these searches did not identify new pathogenicity related genes.

Using the sequenced *O. ulmi* genome, we performed a global domain analysis by searching the entire predicted gene set for protein domains from the Pfam database [41]. In total, 5069 protein domains were found in our *O. ulmi* gene set. Comparison of the protein domains amongst the three fungal species showed that *O. ulmi* has 3993 families in common with *G. clavigera* and 4155 families in common with *N. crassa,* while 605 of the identified domains are found only in *O. ulmi*. However, of those, 205 are domains of unknown function with little information available for them. The remaining 400 unique protein domains are not among those known to play a crucial role in pathogenicity and host-plant cell-wall degradation, such as glycosyl hydrolase, glycosyl transferase and oxidases. Further, we did not observe significant expansion of protein families important in virulence. For instance, the glycosyl hydrolase family is represented by 145 genes in *O. ulmi* compared with 130 genes in *G. clavigera* and 167 genes in *N. crassa*. Overall, our comparison of domain content with *G. clavigera* and *N. crassa* suggests that these three species are very similar. Furthermore, the gene families appear to be highly conserved since no outstanding expansions of protein domain families could be detected in *O. ulmi*.

As another approach to identify virulence factors, we searched for gene models previously known to be associated with pathogenicity in other organisms. To do this, *O. ulmi* gene models were compared against PHI-base [36], a database of 924 fungal-verified virulence and pathogenicity related genes (Table 2)[42,43]. Comparing our results to *G. clavigera* and *N. crassa,* we find that the number of genes from *O. ulmi*, *G. clavigera*, and *N. crassa* with matches to pathogenicity related genes from PHI-base were similar across all three species (610, 598, and 611, respectively); this indicates that there is no obvious expansion in the number of pathogen related genes in *O. ulmi*. While total numbers were similar, a small number of genes from PHI-base represented unique matches for each species (Table 2). These genes are important because they could be responsible for specific characteristics in each species. Three PHI-base genes were found to match only to *O. ulmi* (Table 3). One of these genes, CTB7 from *Cercospora nicotianae*, is annotated as a putative FAD/FMN- or NADPH-dependent oxidoreductase in the cercosporin toxin biosynthetic pathway of *C. Nicotianae*. Cercosporin is a toxin which plays an important role in the pathogenicity of many phytopathogenic *Cercospora* species [44] and may therefore represent an important virulence factor in *O. ulmi.*

**Table 2:** Comparison of the number of PHI-base pathogen genes found in the three species

**Organism**	**Number of pathogen genes (from PHI-base database) found in the organism**	**Number of unique pathogens genes found in the organism**
*O*. *ulmi*	610	3
*G*. *clavigera*	598	2
*N*. *crassa*	611	7

**Table 3:** **PHI-base pathogen genes found in *O. ulmi *not in *N. crassa *and *G. clavigera***

**Pathogen name**	**Description**
PHI:48|CnLAC1|BAD91825|TX:5207|Cryptococcus neoformans|Reduced virulence	A laccase enzyme which catalyzes the synthesis of melanin in the presence of phenolic compounds [43]
PHI:876|MGG_11671|EDK03349|TX:148305|Magnaporthe grisea|Reduced virulence	hypothetical protein similar to reverse transcriptase
PHI:1048|CTB7|ABK64184|TX:29003|Cercospora nicotianae|Reduced virulence	Encodes putative FAD/FMN- or NADPH-dependent oxidoreductases in the cercosporin toxin biosynthetic pathway of C. nicotianae [44]

### Metabolic network reconstruction

With the Pfam and PHI-base analyses indicating overall gene content of *O. ulmi* to be similar to other organisms, we attempted a more detailed analysis of the pathogen’s metabolism. Reconstruction of the metabolic network for *O. ulmi* was achieved by integrating several automated datasets together with ortholog mappings to *S. cerevisae*. In total 1,378 genes (representing 16% of the genome) map to enzymatic activity based on EC number annotation. This number aligns well with the yeast consensus metabolic reconstruction, consisting of 832 genes (representing 13% of its genome) [45]. *O. ulmi* shares 79% (615/783) of its enzymes with *S. cerevisae*.

**Figure 6:**
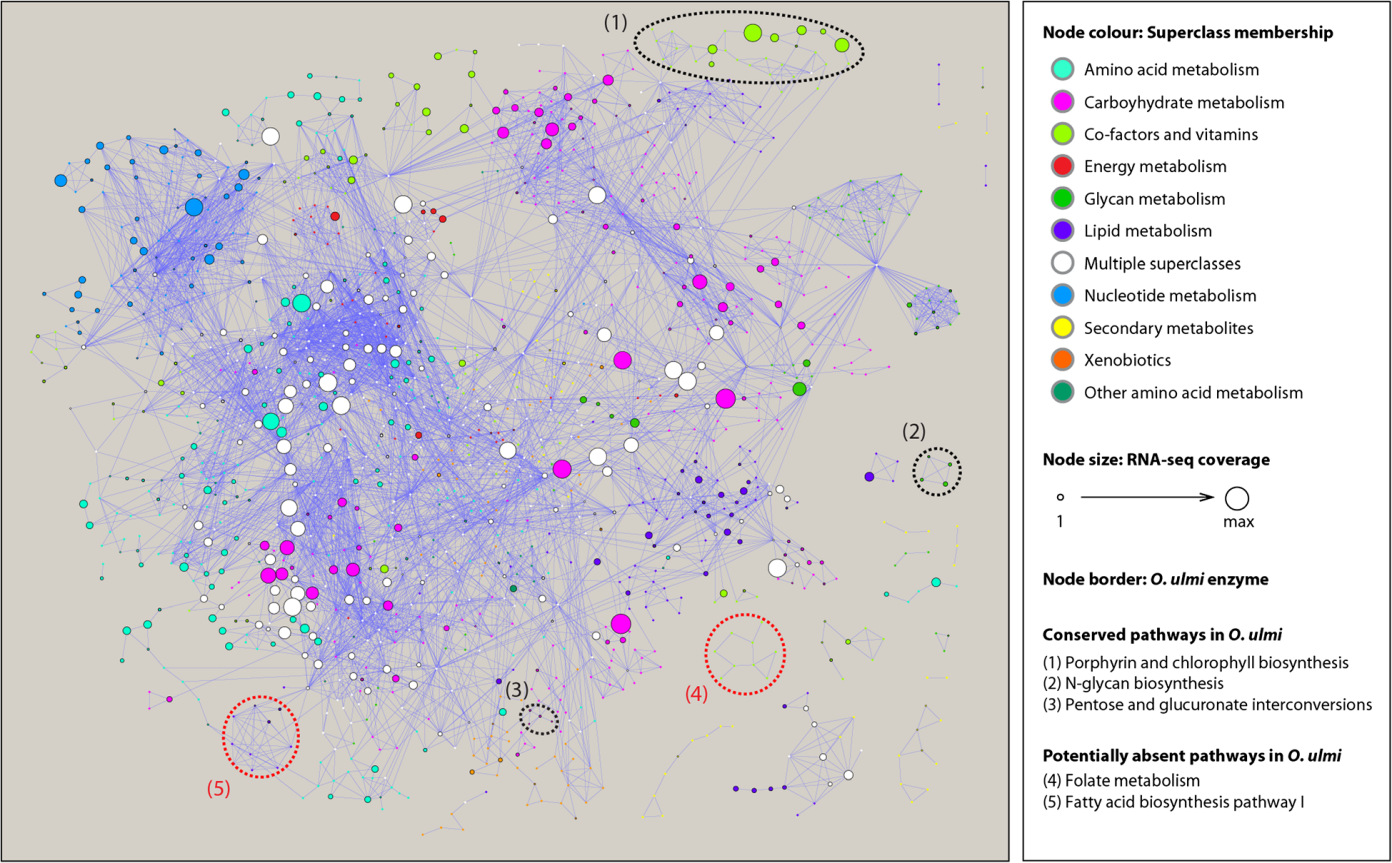
**Metabolic network of *O. ulmi*.** Node size corresponds to RNA-seq expression coverage, with background nodes (borderless) included for context of KEGG pathways. Groups of enzymes dotted-circled in black appear to be conserved in the fungus while enzymes dotted-circled in red appear to be absent in the fungus.

Mapping *O. ulmi* enzymes to KEGG metabolic pathways provides a unique perspective to highlight pathways that are either conserved amongst fungi (represented by yeast) and have been lost in *O. ulmi*, or that are unique to *O. ulmi* and may play a role in causing disease in its host, the elm tree (represented by the model plant genome *A. thaliana*). For instance, pathways for fatty acid biosynthesis and the metabolism of D-arginine and D-ornithine have noticeably fewer enzymes in *O. ulmi* compared to *S. cerevisae* (Figure 6). Other pathways such as anthocyanin biosynthesis and photosynthesis related are plant-specific and as expected appear to be missing from the fungal species. Of interest are a number of pathways that have noticeably greater proportions of enzymes in *O. ulmi* compared to *S. cerevisae* including glycosphingolipid biosynthesis (ganglio series), glycan degradation (N-glycans, gangliosides), lipoic acid metabolism, and drug metabolism by enzymes other than cytochrome P450 (see Figure 6). These activities, particularly those related to glycan degradation, are likely to play an important role in the success and survival of *O. ulmi* as a phytopathogen.

Endopolygalacturonase (ePG) has been identified in *O. ulmi* (DETECT prediction: o7823), and is involved in cell wall degradation. ePG belongs to the polygalacturonase (PG) family of enzymes that catalyze the hydrolysis of pectin compounds which comprise 30% of the primary cell wall in plants [46]. Previous studies have implicated PGs as virulence factors in other phytopathogens including *Botrytis cinerea*[47] and *Alternaria citri*[48] where the enzyme could assist host invasion, tissue destruction and similar processes associated with plant disease. A recent study assessing the role of ePG in *O. ulmi*, however, suggests that the enzyme functions as a parasitic fitness factor as opposed to a virulence factor, given that targeted disruption of the gene led to a reduction in pectin-degrading activity and not a lethal phenotype [49]. Other pectinase enzymes such as pectin methylesterase (BLAST and PRIAM prediction: o5231) and pectinase (BLAST and PRIAM prediction: o3878) present in *O. ulmi* likely act in concert to contribute to successful invasion of the host. The production of PG enzymes is particularly important for the success and survival of *O. ulmi* as it is a pathogen that enters the host directly through a pre-existing wound and therefore lacks specialized penetration structures [50]. Moreover, a role as a minor virulence factor is possible and when combined with other virulence factors, ePG represents a potential target for the control of DED.

In general, core essential pathways such as those related to amino acid, carbohydrate, energy, and nucleotide metabolism are highly conserved across both fungi and plants (see Figure 6). Interestingly, a recent study by Oliveira *et al.*[51] showed that elm trees inoculated with *O. novo-ulmi* had significantly reduced contents of glucose, fructose, starch and sucrose, suggesting that carbohydrate metabolism pathways are important to the pathogenicity of the fungus. Consistent with this hypothesis, the genes encoding carbohydrate metabolism enzymes seem to be highly expressed in *O. ulmi* based on our mRNA-seq data (Figure 6). Moreover, functional categorization of an EST library for *O. novo-ulmi* revealed that the majority of EST sequences associated with metabolism had the greatest representation in carbohydrate metabolism [23]. These results suggest that, while metabolic reconstruction predicts *O. ulmi* has similar enzyme complements for a number of pathways such as those involved in carbohydrate metabolism to *S. cerevisae* and *A. thaliana*, expression profiles are an essential component to assessing the functionality of specific pathways. In addition, the close phylogenetic relationship to *O. novo-ulmi* might also hint that the pathogenic role of *O. ulmi* is at least partially a result of decreasing the plant’s carbohydrates, consequently reducing the efficiency of photosynthesis, and eventually leading to plant senescence.

## Discussion

*Ophiostoma ulmi* caused the first emergence of DED, one of the most destructive plant diseases in the last 100 years [1]. In addition, because of its starch modification characteristics, *O. ulmi* has been used in industry for bioplastic production [21]. However, compared to the more aggressive species *O. novo-ulmi*, little is known about the basic biology of *O. ulmi*.

In this paper, we sequenced the genome of *O. ulmi* using next generation sequencing. Our genome sequence annotation contains 8639 gene models. EST data from the closely related species *O. novo-ulmi*, mRNA-seq data and orthologous genes in other species provide strong evidence for the quality of our annotation. Using genome-scale analysis, we estimated the phylogenetic relationship and distance of *O. ulmi* to *N. crassa* and *G. clavigera*. Finally, we compared the protein domains and matches to PHI-base in our gene models with two other fungal species, *G. clavigera* and *N. crassa* to search for genetic features that may yield important clues about the different lifestyles of the species.

Through metabolic reconstruction, we identified certain families of enzymes that may play a role in the virulence of the fungus. Significantly, we identify a cell wall degrading enzyme, ePG, which may be involved in host-pathogen interactions. The contribution of the gene to virulence has been examined in other fungi, with evidence demonstrating that ePG is required for full virulence and infection of the host tissue [47]. Nevertheless, the full role of the enzyme in pathogenicity for *O. ulmi* has yet to be elucidated.

## Conclusion

Our contribution here was to generate a high-quality genome sequence and annotation for *O. ulmi*. With this in hand, future research will achieve a deeper understanding of the processes by which the fungi colonize and, break down cell wall components and damage elm trees. Furthermore, because of the starch modification and plastic improvement features of the fungus, availability of the fungus genome may help develop new industrial processes for bioplastic production.

## Methods

### Genomic DNA extraction

DNA was extracted from *Ophiostoma ulmi* using Qiagen kit. The fungus was cultured for three days and then after centrifuging the pellet (spores) was washed 3–4 times with distilled water and centrifuged again. The spores were then freeze dried. 500 mg of these spores were crushed using liquid nitrogen and then the powder was suspended in 30 ml of Cell Suspension Solution. 150 μl of Cell lytic solution was then added to it. This was mixed by inverting the tubes 25 times and then incubated at 37 C for 30 minutes. The mixture was then centrifuged and the pellet was suspended in 30 ml of cell suspension solution and 10 ml of Protein precipitation solution. This was mixed by vortex and centrifuged for 3 minutes. The supernatant from this was added to 30 ml of Isopropanol and mixed well. After centrifuging for 1 minute the pellet was washed carefully with 70% Ethanol. This was again centrifuged for 1 minute and then the pellet was air dried. The pellet was then suspended in 5 ml of DNA Hydration Solution and 150 μl of RNase A solution was added and incubated at 37°C for 60 minutes and at 65°C for 60 minutes to dissolve the DNA. This was incubated at room temperature overnight with gentle shaking. The Purity of DNA was evaluated by determining its spectroscopic ratio at A260/A280 nm.

### Genome sequencing and assembly

Genomic DNA was sequenced with Illumina GAIIx as paired-end (PE) reads and mate-paired (MP) reads. Insert size for PE library is ~220 base pairs, and insert size for MP library is ~3000 base pairs. In total we obtained 64,563,784 pairs of PE reads of length 38 base pairs, and 39,690,603 pairs of MP reads of length 40 base pairs. Quality reads are extracted based on the criteria of Illumina pipeline for genome assembly, they are of 86% of the PE reads and 89% of the MP reads. We carried out two rounds of *de novo* assembly of the genome. In the first round, PE and MP reads are trimmed to different lengths and assembled with Velvet [52] separately. For each length several k-mer sizes were tested and the length that gave the maximum N50 was identified. In the second round, the PE and MP reads of the best length from the first round were assembled together with Velvet using several k-mer sizes, and the assembly with the maximum N50 was picked up as the final assembly. There are 3,415 contigs in the final assembly, the largest contig contains 3,256,915 base pairs, and total base pairs of all contigs are 31,466,092. N50 of the final assembly is 1,009,735 base pairs. 164,295,551 reads out of 180,796,760 total reads were used in the final assembly.

### Gene prediction methods

*Ab initio* gene prediction has been done using GeneMark-ES-v2.0 [28] with default parameters. We used this method for two reasons. First, it is designed specifically for fungus genome sequences. In addition, unlike other gene prediction methods, it does not have the bottleneck of a large training set to train their underlying model. It computes the model parameters from the genome sequence. We aligned the protein sequences from the other species *G.clavigera* and *N.crassa* to *O. ulmi* sequences using Exonerate [29]. For protein comparison, the protein2genome model was used and the bestn parameter was set to 1 to find the best matches to the protein sequences. However, Because of the possibility of gene duplication and gene expansion we also included the genes predicted using bestn 10 parameter which were not overlapping with bestn 1 gene models. Our final gene model set was the combination of the genes predicted by *ab initio* and comparative gene predictors. Initially the set comprised of the genes predicted by *ab initio* gene predictor and then the genes that are obtained from comparison with *G. clavigera* and *N. crassa* protein sequences were added to the set if they do not overlap the initial gene set.

### mRNA-seq

*O. ulmi* was grown and harvested under similar condition described above for its genomic DNA extraction. Total RNA isolation was carried out using a Qiagen RNA preparation kit (Qiagen Inc., Mississauga, ON, Canada) by following the supplier instructions for filamentous fungi. cDNA was synthesized at CAGEF using mRNA-seq sample preparation kit following the supplier instructions (Illumina Inc., San Diego, CA). *O. ulmi* mRNA was sequenced with Illumina GAIIx as paired-end (PE) reads as described above. We had approximately 93 million paired-end reads of length 38. In order to evaluate the quality of predicted genes, we mapped the reads back to the genome sequence using Bowtie.0.12.7 [11]. The bowtie-build command was used to build an index from the genome sequence and then we ran Bowtie using bowtie command with default parameters. Approximately 30% of the mRNA-seq read data was mapped to the genomic DNA sequence. The rest of the read data were of low quality and could not be aligned to the sequence. Using the coordinates of mapped reads, the overall average coverage and the coverage for the coding regions of each gene was calculated by dividing the total length of the reads by the total number of base pairs for every desired region.

### Phylogenetic analysis

We found orthologous groups among four species *O. ulmi*, *G. clavigera*, *N. crassa* and *S. cervisiae* using Inparanoid_4.1 [30] with the default settings. First the protein sequences for all the four fungi were searched against each other using BLAST with the default parameters and then the orthologous groups were identified between every two species. We employed these pairwise ortholog groups in building files which contains four gene models each from one of the species and the gene models were pairwise orthologs. This resulted in 2215 files. Then we aligned the gene models for each file using the multiple sequence aligner MAFFT [53] and the phylogenetic analysis was performed with PAML [31]. The parameters for running PAML were as follows: Empirical amino acid substitution model and removing gaps columns. For each alignment we computed the likelihood of four trees: tree 1 (((*O. ulmi*, *G. clavigera*), *N. crassa*), *S. cerevisiae*), tree 2 ((*O. ulmi*, (*G. clavigera*, *N. crassa*)), *S. cerevisiae*), tree 3 ((*O. ulmi*, *G. clavigera*, *N. crassa*), *S. cerevisiae*) and tree 4 (((*O. ulmi*, *N. crassa*), *G. clavigera*), *S. cerevisiae*). 1926 of the alignments (86%) support the tree 1 with 90% bootstrap support cutoff.

### Metabolic reconstruction

The gene model for *O. ulmi*, containing 8, 639 genes, was searched against the SwissProt-Uniprot protein database (v 58.0) using the following homology-based enzyme prediction tools: (i) DETECT [54] (cutoff ILS > 0.2, at least 5 positive hits), (ii) BLAST (E-value > 1e-10), (iii) PRIAM [55] (E-value > 1e-10), and (iv) ortholog mappings to Yeast based on OrthoMCL [56,57]. No pathway data for *Ophiostoma* was available from KEGG. The BRENDA resource (Barthelmes, *et al.*, 2007) provided biochemical evidence for four enzymes. The final set of 783 enzymes from *O. ulmi* was obtained by integrating the datasets from BRENDA, DETECT, Yeast orthologs, and enzymes identified by both BLAST and PRIAM. See (data on our website) for gene-EC mappings and for corresponding evidences. Yeast and *Arabidopsis thaliana* EC numbers were obtained by combining species-specific datasets from BRENDA and BioCyc (YeastCyc and AraCyc, respectively).

### Pathway heatmap

Ratios of enzyme complements from *O. ulmi*, Yeast, and *A. thaliana* were calculated based on KEGG pathways and grouped according to superclass. Note that KEGG incorporates information for all species available, so many pathways may include enzymes that are not relevant leading to misleading interpretations of pathways that might appear absent or present in the species.

### Data availability

The sequence and annotation data are available at (http://www.moseslab.csb.utoronto.ca/o.ulmi). These include genome sequence, datasets for genes and proteins, a summary of the results from Pfam analyses and a Blast server.

## Competing interests

The authors declare that they have no competing interests.

## Authors’ contributions

SK (Shima) drafted the manuscript, carried out genome annotation and bioinformatics analyses. SH reconstructed the metabolic network, participated in drafting the manuscript. SK(Sadia), prepared RNA from *O. ulmi*. YG performed genome assembly, participated in drafting the manuscript. VT prepared genomic DNA from *O. ulmi*, participated in drafting the manuscript. JP supervised SH. MS co-supervised SK(Sadia) and VT. AMM supervised SK(Shima) and edited the manuscript. DC co-supervised SK(Sadia) and VT, interpreted the sequencing data and prepared the manuscript. All authors read and approved the final manuscript.
